# Sources of Traffic and Visitors’ Preferences Regarding Online Public Reports of Quality: Web Analytics and Online Survey Results

**DOI:** 10.2196/jmir.3637

**Published:** 2015-05-01

**Authors:** Naomi S Bardach, Judith H Hibbard, Felix Greaves, R Adams Dudley

**Affiliations:** ^1^University of California San FranciscoDepartment of Pediatrics, Philip R Lee Institute of Health Policy Studies, Center for Healthcare ValueSan Francisco, CAUnited States; ^2^University of OregonHealth Policy Research Group ISEEugene, ORUnited States; ^3^Harvard School of Public HealthDepartment of Health Policy and ManagementCambridge, MAUnited States; ^4^University of California San FranciscoPhilip R Lee Institute of Health Policy Studies, Center for Healthcare ValueSan Francisco, CAUnited States

**Keywords:** consumer health information, Internet/statistics and numerical data, search engine, quality of health care, consumer behavior

## Abstract

**Background:**

In the context of the Affordable Care Act, there is extensive emphasis on making provider quality transparent and publicly available. Online public reports of quality exist, but little is known about how visitors find reports or about their purpose in visiting.

**Objective:**

To address this gap, we gathered website analytics data from a national group of online public reports of hospital or physician quality and surveyed real-time visitors to those websites.

**Methods:**

Websites were recruited from a national group of online public reports of hospital or physician quality. Analytics data were gathered from each website: number of unique visitors, method of arrival for each unique visitor, and search terms resulting in visits. Depending on the website, a survey invitation was launched for unique visitors on landing pages or on pages with quality information. Survey topics included type of respondent (eg, consumer, health care professional), purpose of visit, areas of interest, website experience, and demographics.

**Results:**

There were 116,657 unique visitors to the 18 participating websites (1440 unique visitors/month per website), with most unique visitors arriving through search (63.95%, 74,606/116,657). Websites with a higher percent of traffic from search engines garnered more unique visitors (*P*=.001). The most common search terms were for individual hospitals (23.25%, 27,122/74,606) and website names (19.43%, 22,672/74,606); medical condition terms were uncommon (0.81%, 605/74,606). Survey view rate was 42.48% (49,560/116,657 invited) resulting in 1755 respondents (participation rate=3.6%). There were substantial proportions of consumer (48.43%, 850/1755) and health care professional respondents (31.39%, 551/1755). Across websites, proportions of consumer (21%-71%) and health care professional respondents (16%-48%) varied. Consumers were frequently interested in using the information to choose providers or assess the quality of their provider (52.7%, 225/427); the majority of those choosing a provider reported that they had used the information to do so (78%, 40/51). Health care professional (26.6%, 115/443) and consumer (20.8%, 92/442) respondents wanted cost information and consumers wanted patient narrative comments (31.5%, 139/442) on the public reports. Health care professional respondents rated the experience on the reports higher than consumers did (mean 7.2, SD 2.2 vs mean 6.2, SD 2.7; scale 0-10; *P*<.001).

**Conclusions:**

Report sponsors interested in increasing the influence of their reports could consider using techniques to improve search engine traffic, providing cost information and patient comments, and improving the website experience for both consumers and health care professionals.

## Introduction

There is unprecedented interest in making information about provider cost and quality of care publicly available. The Affordable Care Act (ACA) expands coverage to millions, with variable levels of deductibles, leading to increased demand from consumers for cost and quality data [1,2]. In addition, the ACA requires insurance exchanges to create websites that provide comparative health plan performance on quality and cost [3]. As physician performance metrics become publicly available under the ACA’s Value-Based Payment Modifier program [4], the hope is that public reporting will drive provider choice and stimulate greater quality improvement efforts among providers to a degree not seen before. And yet, consumers have been slow to use this information to inform choices [5-7]. However, there is evidence that well-designed reports of quality can influence consumers to choose higher quality providers [8]. Because public reporting has the potential to improve quality, multiple stakeholders are interested in understanding how best to reach consumers and how to provide relevant quality information [1]. This study aims to add to that understanding.

Prior work about the users of public reports of quality has focused on specific user segments (eg, clinicians or patients) and predictors of consumer use. Some clinicians use public reports for internal quality improvement [9,10]. A small proportion of US consumers report seeing comparative hospital or physician quality information, although that number appears to be increasing [5,6]. Studies of sociodemographic predictors of physician rating websites for German consumers were mixed regarding whether education, age, gender, or chronic disease predicted use and awareness of the physician rating websites [11-13].

Despite knowledge that people find online information through a variety of routes, there is no peer-reviewed literature of which we are aware about how people find public quality and cost reports (eg, via search engines searches, links on other websites, direct emails) or whether specific traffic sources are associated with overall traffic. In addition, there has been no information gathered in real time from US online visitors to the reports, which limits our understanding of what is relevant to users as they interact with the reports. Lastly, prior work has not described differences in website experience for different visitors (eg, consumers compared to physicians), not their areas of interest. Because the influence of these reports depends on who finds and uses them, improving the reports’ impact will be difficult without understanding how reports currently garner an audience and without knowing the needs of consumer and physician visitors who find the reports.

In order to address this knowledge gap, we used 2 types of data gathered from a group of public reporting websites of hospital or outpatient provider quality: Web analytics data that passively tracks website visitor traffic and behavior, and an online survey of website visitors. We describe overall traffic to the sites, how visitors arrive at the websites, differences between the sites in traffic sources and types of visitors (eg, consumers vs physicians), visitors’ purposes in going to the website, and their experience while there. The objective of this study is to inform transparency efforts by assessing for potential ways to increase traffic to online public reports of provider-level quality and cost as well as meet the needs of the visitors who find them.

## Methods

### Setting

The Learning Network of Chartered Value Exchanges (CVEs) is a program that has supported transparency since 2004 and is sponsored by the Agency for Healthcare Research and Quality (AHRQ). The CVEs are multistakeholder state or local quality collaboratives that are investing significant resources in online public reports of hospital and physician quality in their communities. The CVEs involve more than 550 health care leaders and represent more than 124 million lives, more than one-third of the US population [14]. Because of the CVEs’ long experience and broad catchment area, we used the network of CVEs for this study.

All 24 CVE’s were invited to participate, with 22 CVE or CVE-affiliated websites active. Of these, 18 websites participated. The websites were public quality reports of hospitals, outpatient providers (medical groups or clinics), or both. Websites reported on providers within a state, a region in a state, or a county. Quality measures for hospitals were commonly Center for Medicaid and Medicare Services (CMS) measures, although some websites also included measures of maternity and neonatal care. Quality measures for outpatient providers were often Healthcare Effectiveness Data and Information Set (HEDIS) measures and Consumer Assessment of Healthcare Providers and Systems (CAHPS) patient experience measures. Narrative patient comments were not shown on any participating website.

Further descriptions of CVE groups and websites are available [14]. Participating websites agreed to participate on the condition that we not identify the websites and their performance individually. Multimedia Appendix 1 shows a screenshot from one of the websites at the time of the study, whose sponsors gave permission to share it.

### Website Analytics Data Collection and Search Term Coding

We gathered Web analytics data from February to August 2011 using Google Analytics [15]. We excluded data from the IP addresses of computers of the report sponsor organization and of any external vendors hosting the online reports.

Collected variables were number of unique visitors and percent of unique visitors arriving via 3 methods: search engine queries (“search traffic”), clicking on a link from a different website (“referral traffic”), or directly entering a URL into a Web browser or clicking on a link in an email, word processing document, or document in Portable Document Format (PDF) (“direct traffic”).

The population density varied among the catchment areas the websites served. To generate population-adjusted website traffic from the absolute number of unique visitors, we used Census Bureau catchment area counts of 100,000 Internet-using households as the denominator under total number of unique visitors to calculate “per capita traffic” to each website [16,17].

We also collected search terms (eg, “best doctors San Francisco”) for all search engine traffic. The primary author (NSB) and a research staff member (RAP) organized the search terms into categories using an iterative process: both investigators read the 50 most common search terms for each website then discussed the various categories of search terms to create a codebook for categories. RAP then coded the top 50 search terms for each website and NSB reviewed the initial coding and discussed any code changes. They then combined codes into larger groupings for the final analysis. The codebook and documentation of the coding process is available on request.

We could not link the Web analytics data to survey responses because the analytics data do not include visitor IP addresses and because they are reported in aggregate.

### Survey Development and Content

The primary aim of the survey was to provide information on report visitors’ use and perceptions of the value of the public reporting websites. See Multimedia Appendix 2 for the full Checklist for Reporting Results of Internet E-Surveys (CHERRIES) checklist for reporting of Internet e-surveys and for additional description of survey development [18]. We drafted the AHRQ Public Report [19] surveys based on the authors’ expert knowledge about online public reports and drawing from existing surveys that participating public reporting websites were using.

Survey items fell into the following categories: demographics, purpose of the visit, medical topics of interest, and areas of quality measurement of interest. Visitors were shown questions within each topic tailored to their persona type (eg, patient, friend or family member, health care professional, insurer, employer or labor union, researcher, media, lawyer, legislator). We focused on the results for the consumers (patients or friends or family members) and health care professionals in this paper because these were the largest groups of respondents and are the ones most likely to use the reports for choice or for performance improvement. The surveys are available in Multimedia Appendix 2.

### Survey Data Collection and Response Coding

We surveyed participating website visitors from February to August 2011. We used an “open survey” design in which all visitors viewing at least 1 page with access to quality performance measures were offered the opportunity to take the survey. The invitation appeared in a pop-up window with directions to take the survey at the end of the session. See Multimedia Appendix 1 for images of invitation and survey.

We framed the survey respondents as a group interested enough in the website content to spend time on an online survey afterward. The implication is that although there might be a low response rate, the responses we received would be from people who are more likely to be potentially influenced by the report. We adopted this frame because low response rates are a known limitation for website surveys because a proportion of website visitors are searching for other content or have limited attention or time for a survey while online. For instance, Kaiser researchers had a 17% response rate in an online survey of users of a secure online personal health record who were presumably more engaged than one-time visitors to a website [20]. One approach to the known low response rate is to invite only visitors who interact extensively with a website to answer a survey, thus creating a smaller response rate denominator. We chose to invite all visitors because some websites have quality information on only 1 or 2 pages.

To decrease response burden, we programmed the survey software to show each consumer (patient or friend/family member) a randomized set of 3 of 5 item groupings (eg, purpose of visit, demographics, topics of interest), leading to smaller consumer sample sizes for each set of questions than if all consumers had answered all items. As noted in our results, the denominators for each of these item sets only included those who were randomized to see those questions.

We coded free-text survey responses using the existing survey item options and categorizing responses that did not fit into an existing option as “other.” We allowed a new category to be formed if it occurred more than 10 times and put the answers for an existing option into “other” if the option was chosen less than 10 times. The only option affected was the consumer primary purpose of visit, for which “learn about a disease” was included in “other.”

### Statistical Analysis

#### Analysis of Traffic to the Websites

We assessed 2 potential predictors of per capita traffic on the websites. We used the same approach in 2 separate models and websites were the unit of analysis. The predictors were the percent of visitors to the website that were from search engines and, for the websites with >15 survey respondents, the percent of consumer respondents to the survey. We estimated linear regression models. In each model, we included a term for report type (hospital only, physician only, or both), assuming that reports with both types might have higher traffic. We performed sensitivity analyses allowing for clustering by website and using a binary variable for report type (1 provider type vs both provider types).

#### Analysis of Survey Responses

We calculated response rate statistics: view rate (unique visitors shown the survey/all unique site visitors) and participation rate (number of surveys with at least 1 question answered/unique visitors shown the survey) [18]. See Multimedia Appendix 2 for response rate analysis details. We used a t test to compare health care professional and consumer website experience scores. We used chi-square tests to compare health care professional and consumer primary purposes of visit and other areas of interest. As a sensitivity analysis, to assess whether the associations differed by report type (physician or hospital report), we performed the same analyses using survey responses stratified according to whether the survey was answered from a hospital or a physician reporting page.

All analyses were conducted using Stata 12 (StataCorp LP, College Station, TX, USA). The University of California San Francisco Committee on Human Research approved this study.

## Results

### Website Analytics Data on Volume and Sources of Visitors

For the 18 websites, there was an average of 1440 unique visitors per month per website, with a total of 116,657 unique visitors to all websites. There was substantial variability in website per capita traffic (range 1-167 unique visitors/100,000 Internet users per month; median 31.8, IQR 15.7-47.2).

The websites commonly reported on hospital quality (89%, 16/18), with 61% (11/18) also reporting on clinic or medical group quality (Table 1). There were fewer reports from the Southern region compared to other regions, with reports approximately evenly split through the rest of the regions. Most reports had a state as a catchment area, with all websites reporting at the provider level (hospitals, clinics, or medical groups).

**Table 1:** Characteristics of participating websites (N=18).

Characteristics		Websites, n (%)
**Public report type**		
	Hospital	7 (39%)
	Clinic or medical group	2 (11%)
	Both	9 (50%)
**Region**		
	West	5 (27%)
	Northeast	5 (28%)
	Midwest	6 (33%)
	South	2 (11%)
**Catchment area**		
	State	13 (72%)
	County	5 (27%)
**Number of per capita monthly unique visitors** ^a^		
	1-20	7 (39%)
	21-50	6 (33%)
	>50	5 (28%)

^a^ These are the numbers of unique visitors/100,000 Internet users in the catchment area arriving at the websites per month.

**Figure 1:**
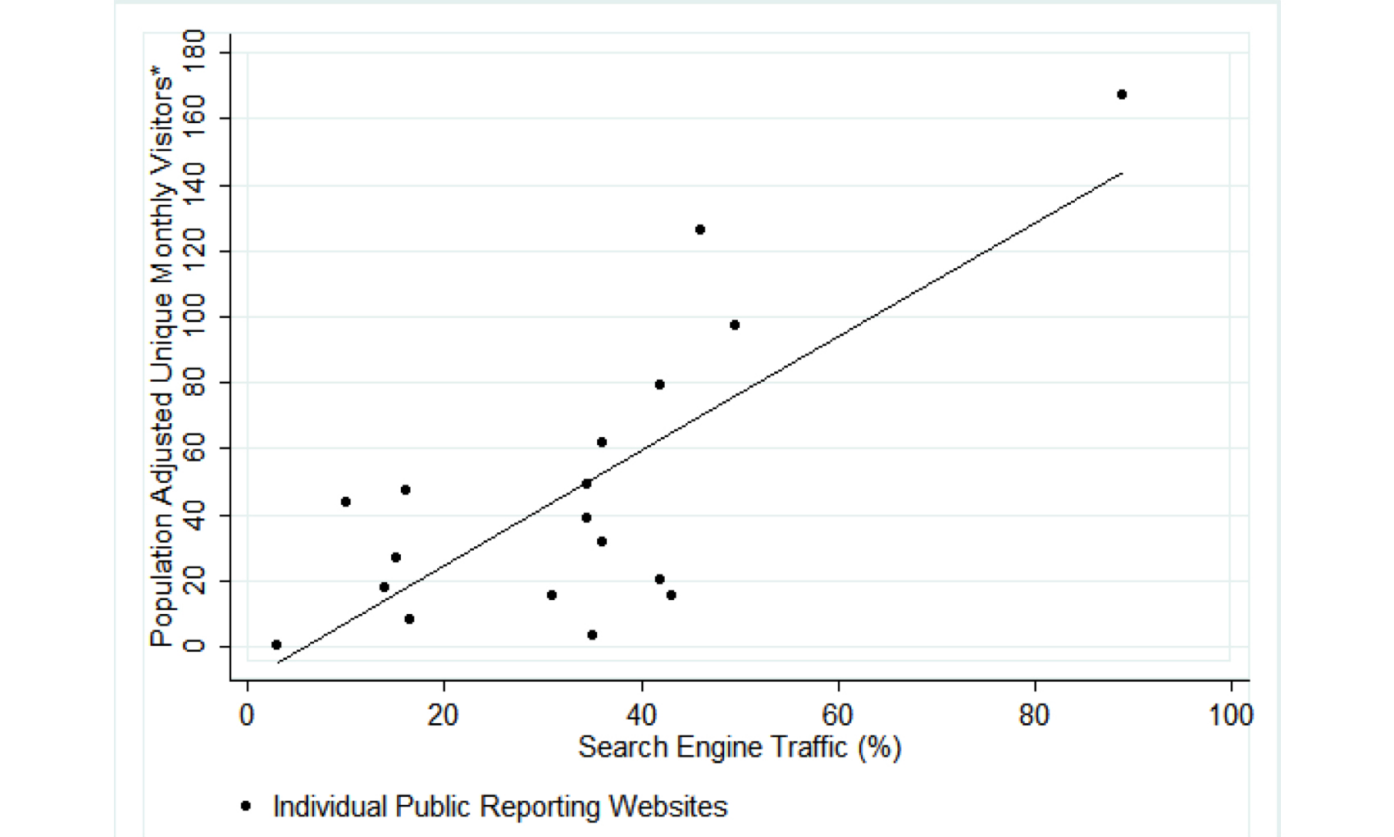
Relationship between proportion of traffic from search engines and population-adjusted number of unique monthly visitors to public reporting websites of hospital and outpatient provider quality. *This is the per capita traffic: the number of unique monthly visitors per 100,000 Internet-using households in the catchment area of the individual public reporting website.

Visitors arrived most often through a search engine query (63.95%, 74,606/116,657 of unique visitors) (Table 2) and less often through referral from another website (15.80%, 18,432/116,657) or direct links received in an email or in an electronic document (19.99%, 74,606/116,657). There was a positive association between percent of unique visitors arriving from search engines and total unique monthly visitors per 100,000 Internet-using households in the catchment area, adjusted for report type (hospital only, outpatient group only, or both), with a 1-point increase in traffic for every 1.8% point increase in proportion of search traffic (*P*=.002) (Figure 1). The sensitivity analysis that allowed clustering by website returned similar results.

**Table 2:** How visitors arrived at the websites and categories of search terms used by those arriving via search engines (N=116,657 unique visitors).

Traffic sources		Total traffic, n (%)
**Route of arrival (N=116,657)**		
	Direct traffic	23,331 (20.00%)
	Referral traffic	18,432 (15.80%)
	Search engine traffic	74,606 (63.95%)
**Search terms used (n=74,606)**		
	Hospital name	27,122 (23.25%)
	Website name	22,672 (19.43%)
	Website to compare providers	15,998 (13.71%)
	Other	4988 (6.69%)
	Medical condition	605 (0.81%)

^a^Direct traffic arrives by directly entering the website URL into the browser or by clicking on a link in an email, word processing document, or PDF document. Referral traffic arrives at the websites through clicking on a link from a different website. Search engine traffic arrives via Web search engines (eg, Google, Yahoo, or Bing).

^b^“Search terms used” refers to phrases or words used by search engine traffic visitors (eg, “best doctors XXX city”). “Website to compare providers” refers to search terms for hospital comparison such as “best hospitals in Maine.”

For most websites (61%, 11/18), the search term by which visitors arrived most often was the name of the website. However, collectively, searches for individual hospitals by name were the bulk of the searches that led to visits across all participating websites (Table 2). Among the 2 websites with the highest traffic, responsible for 56.55% (65,967/116,657) of all unique visitors, the hospital name was the most common search term (37.00%, 17,205/46,500 and 97.23%, 9891/10,172 of search terms used), and search was the most common source of traffic (89.00%, 46,500/52,247 and 74.14%, 10,172/13,720 of unique visitors, respectively).

### Survey Data on Consumer and Health Care Professional Visitors

Of all unique visitors (N=116,657), 49,560 were presented the option to take the survey, resulting in a view rate of 42.48%. Of those who viewed the invitation, 1755 responded, resulting in a participation rate of 3.54%. The number of responses from the websites ranged from 2-287 (mean 97.5, SD 98.6; median 49.5, IQR: 26-143).

**Figure 2:**
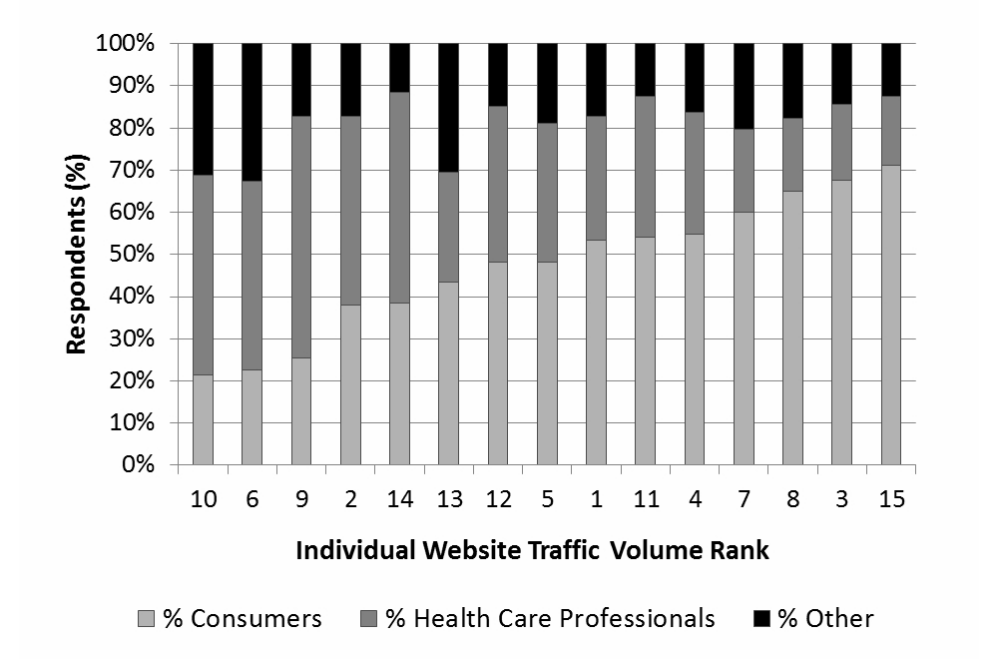
Variation in proportions of consumer and health care professional respondents across websites.

There were more consumer respondents (850/1755, 48.43%) than health care professional respondents (551/1755, 31.39%). The remaining respondents were members of the media, employers or labor union members, researchers, insurers, or others who chose a free-text option (354/1755, 20.17%). There was wide variation across websites in proportions of respondents who were consumers (21%-71%) and health care professionals (16%-48%). Figure 2 displays that variation and shows ranking according to per capita traffic. Figure 2 illustrates that there is no association between audience composition and website traffic (*P*=.56 for regression of proportion of consumers and per capita traffic, with similar results in the clustered sensitivity analysis).

More health care professionals than consumers had a primary purpose of choosing or comparing providers (38.8%, 168/433 vs 25.3%, 108/427) (Table 3), whereas more consumers than health care professionals (27.4%, 117/433 vs 20.3%, 88/427) had a primary purpose of finding quality information on a specific provider (*P*<.001 for overall comparison). For consumers with a primary purpose to “choose providers” who were also asked whether they did so (n=51), 78.4% (40/51) were likely or very likely to use the information to choose a provider. Only 4.2% (18/433) of health care professionals said that they came to the websites for the purpose of patient referral to a hospital or other health care provider (Table 3). Sensitivity analysis found that these patterns were similar by report type (data not shown).

Few providers (0.9%, 4/433) or consumers (2.6%, 11/427) had a primary purpose of looking at cost information. The interest in this information was more common, with both providers (26.6%, 115/433) and consumers (20.8%, 92/442) desiring cost content to be added to the websites (Table 3). Approximately one-third of consumer respondents indicated interest in adding measures about diseases relevant to them (36.4%, 161/442) or adding written comments from other patients (31.5%, 139/442). Consumer respondents rated their experiences using the website lower than did health care professional respondents (mean 6.2, SD 2.7 vs mean 7.2, SD 2.2 on a scale 0-10, *P*<.001).

Sensitivity analysis found that these differences in primary purpose by respondent type and in mean experience scores were similar in analyses stratified by report type (hospital report vs outpatient quality report, data not shown).

Consumer respondents were commonly middle aged (58.1%, 194/334 were 45-64 years), white (84.4%, 217/257), and many had private insurance (74.1%, 238/321). Additional respondent characteristics are in Multimedia Appendix 3.

**Table 3:** Comparison of health care professionals’ and consumers’ primary purposes for visits to public reports and overall website experiences.

Survey topics		Consumers	Health care professionals
**Primary purpose, n (%)** ^a^			
	Choose or compare providers^b^	108 (25.3)	168 (38.8)
	Find quality information on a specific provider^c^	117 (27.4)	88 (20.3)
	Cost information	11 (2.6)	4 (0.9)
	Referral to another provider	—^h^	18 (4.2)
	Get practical information^d^	25 (5.9)	20 (4.6)
	General interest in website content	10 (2.3)	26 (6.0)
	Other^e^	68 (15.9)	52 (12.0)
	No answer	88 (20.6)	57 (13.2)
Overall website experience,^f^ mean (SD)		6.2 (2.7)	7.2 (2.2)
**Additional content endorsed,** ^g^ **n (%)**			
	Measures about other diagnoses, relevant to their condition	161 (36.4)	—^i^
	Patient comments	139 (31.5)	—^i^
	Practical information^d^	48 (10.9)	—^i^
	Additional providers	45 (10.2)	43 (9.9)
	Costs	92 (20.8)	115 (26.6)
**Additional measures, n (%)**		—^h^	99 (22.9)
	More methods (eg, risk adjustment model)	—^h^	113 (26.1)
	More detailed results (eg, 95% CIs)	—^h^	112 (25.9)
	Physician-level data	—^h^	81 (18.7)

^a^ *P*<.001 for differences between categories of Primary purpose by persona. Consumers n=427; health care professionals n=433.

^b^ This could be choosing a provider for oneself or a friend or family member. For providers, this included comparing oneself to other providers.

^c^ For providers, this included looking at one’s own performance only.

^d^ For example: address, hours of operation, services available.

^e^ Only 2.1% (7/427) of consumers chose the primary purpose of “learn about a disease” and so it was included in “other.”

^f^ *P*<.001 for difference in overall experience on website between health care professionals and consumers. Consumers: n=697; health care professionals: n=499.

^g^ Respondents could choose more than 1 answer leading to total percentages >100%. Consumers: n=442.

^h^ These options not presented to consumers.

^i^ These options not presented to health care professionals.

## Discussion

### Principal Findings

In this study of Web analytics data and real-time survey data from a multistate group of public reports of quality, we analyze variations in sources of traffic across websites as well as audience type and purpose. We found that overall traffic to the sites is low. Per capita Internet traffic varied extensively across sites, with higher traffic on websites associated with higher proportion of traffic from search engines. Most visitors arrived after a Web search, frequently using search terms for a specific hospital. Although both consumers and health care professionals use the websites to assess provider quality and choose a provider, websites varied substantially in the proportion of consumer or health care professional respondents, and consumer respondents had a less positive experience on average than health care professionals. Our findings speak to 2 mechanisms through which a public report can be influential: achieving a larger audience and meeting the needs of the audience that arrives.

### Achieving a Larger Audience

The number of visitors to these websites was low (1440 unique visitors/month) compared to a similar site in the United Kingdom, the NHS Choices website, which posts numerical ratings of hospitals and physicians as well as written comments from patients. NHS Choices has 250,000 page views per month (some of which could be repeat visitors) to the pages that show comparative provider performance [21]. To the extent that public reports influence choice [22], leading to better health, or that public reporting can incentivize providers to improve care [23,24], increasing the use of US public reporting websites will be important. The UK experience implies that it is potentially possible to do so.

In our group of websites, the range of per capita traffic was large, with the 2 websites with the highest per capita traffic achieving most of their traffic via search queries. This, taken with the finding that search traffic was >60% of traffic overall and that a higher proportion of search traffic is statistically significantly associated with higher overall traffic, suggests that increasing search engine traffic is a potentially powerful approach to increase traffic overall. This is supported by data that in 2012-2013 there were 97,000 monthly Google searches using keywords related to hospital quality, suggesting that consumers are actively searching for this information [25].

We also found that very few visits to the websites came from searches for specific diseases. A similar finding in the survey was that few consumer respondents chose “learn about a disease” as their primary purpose for their visit. Taken together, these findings imply that the provision of disease-specific information only (eg, sections titled “learn about your diabetes” without performance measures) may not increase traffic to the site.

Lastly, we found that the 2 websites with the highest search traffic and the highest traffic overall had hospital names as the most common search terms leading to their visits. These websites had also “tagged” (ie, made visible to search engines) the hospital names, whereas the other websites did not. Hence, our data suggest that tagging provider names on a public reporting website may increase the chances of the website being found in a search and lead to more visitors.

### Visitor Experience and Preferences

The influence of a public report also depends on the types of people who find the report and whether the content meets their needs. Prior work in this area includes assessments of predictors of reported past use of online physician rating websites and willingness to pay for physician rating sites, examining consumer demographics as well as models of underlying factors motivating consumer use of physician rating websites (digital literacy, perceived ease of use of Internet) [12,26,27]. Our findings add to this work by surveying visitors in real time, including visitors to hospital public reporting websites, assessing physician and consumer perspectives separately, and assessing the purpose of website visitors and their areas of interest. These new contributions may be useful to report sponsors who would like to not only understand predictors of use, but also preferences of their current audience in order to meet their needs. If the traffic on these sites grows, report sponsors or researchers could track whether the user composition changes and whether audience preferences change.

We found that consumer survey respondents on these websites were predominantly older, white, and privately insured. The high proportion of privately insured respondents could be due to low response rate among other respondents or due to low numbers of uninsured or publicly insured visitors. Should the latter be true, one implication is that vulnerable populations who have historically received lower quality of care may access quality information for provider choice less often. Prior research found that African American survey respondents were less likely than white survey respondents to report seeing comparative quality information for hospitals and doctors [28], even though once minorities are aware of reports, they are more likely to use the information [28,29]. To the degree that use of quality information drives health outcomes, disseminating the information to vulnerable populations, as some communities are already trying to do [30], is one approach to avoid widening existing disparities. Additional research is needed regarding whether vulnerable populations have a choice of provider and, if so, the most effective way to disseminate and encourage the use of the quality information to this group [11].

Websites vary in the proportion of consumer and health care professional respondents. Berwick and Coye [31] and Hibbard [9] describe differences in consumer and health care professional pathways through which quality reports may stimulate improvement; hence, both audience types are important. Future research could assess for potential differences between the marketing approaches or website features to explain differences in report visitor composition. We did not find a relationship between the proportion of consumer respondents and per capita traffic, implying that the higher traffic sites are not necessarily succeeding in the consumer populations. Our findings on website experience suggest that additional research is needed to improve the experience for both audiences. Our data show that although there is some overlap in interests between consumers and health care professionals, the consumer website experience was worse than the experience of health care professionals. The needs of health care professionals and consumers may be different enough that a single report will not be adequate to effectively drive quality improvement through the separate pathways Hibbard and Berwick and Coye describe, but additional research is sorely needed in this area.

Few survey respondents indicated that the primary purpose of their visit was to obtain cost information, likely reflecting that most websites lacked cost information. However, 21% of consumers and 27% of health care professionals indicated interest in adding cost information. Since we collected our data, the insurance market has evolved with greater use of high deductible plans and more cost shifting to consumers. In this context, consumer interest in costs may increase [1], especially if there is increased awareness of variation among providers in cost of care [32]. Our data suggest that providing cost information on the websites may be an opportunity to meet the needs of visitors to the public reports.

Consumer respondents reported an interest in seeing patient comments. This is similar to prior literature showing that consumers find patient stories to be as persuasive as quantitative assessments of patient experience [33]. Providing patient comments on provider quality may better meet the needs of consumer visitors. Additional research is needed to understand how to best elicit narrative comments and how to display narrative and quantitative data together to facilitate optimal choices [8,11].

### Limitations

The survey participation rate was low (3.6%) leading to a risk of nonresponse bias. This is a known limitation for website surveys in real time [20] and the results represent the only available data from recent visitors to online public reports. The nonresponse bias in our survey might be particularly relevant in the younger age groups who are poorly represented in these data and are often poor responders to Internet and other types of surveys [34]. This is supported by our findings in Multimedia Appendix 3 showing that the consumer respondents were older than the population in the catchment areas who reported using the Internet to research health plans or practitioners in 2011. Additional methods to gather data from consumer groups who do not answer surveys may be necessary to complete our understanding of visitors to public reports. Response bias does not affect the Web analytics data.

### Conclusions

Under the ACA, there is new support for health care transparency and a unique opportunity to help consumers choose higher quality providers. If public reports of provider performance and cost are to be effective, consumers and health care professionals need to find them and visitors to the sites need to find what they need there. These new data suggest that online performance reports of physicians and hospitals are not frequently found and, when found, that the website experience can be improved for both health care professionals and consumers. Using specific search engine techniques may garner a larger audience. Developing reports that cover a broader set of medical conditions, that include patient comments, or that provide cost information could enable website sponsors to better meet visitors’ needs.

## Multimedia Appendix 1

Example of a comparative online public report of hospital quality and screenshots of the survey invitation and an example survey question.

## Multimedia Appendix 2

(1) CHERRIES Checklist for Reporting Results of Internet E-Surveys; (2) Description of Survey Development; (3) AHRQ Hospital-Public Report Survey; (4) AHRQ Physician-Public Report Survey; (5) Response Rate Description.

## Multimedia Appendix 3

Description of website survey respondents.

## Abbreviations

ACA: Affordable Care Act
AHRQ: Agency for Healthcare Research and Quality
CAHPS: Consumer Assessment of Healthcare Providers and Systems
CVE: Chartered Value Exchanges
HEDIS: Healthcare Effectiveness Data and Information Set
PDF: Portable Document Format
