# Supplementary figures and images for: Sources of Traffic and Visitors’ Preferences Regarding Online Public Reports of Quality: Web Analytics and Online Survey Results
Source: J Med Internet Res. 2015 May 1;17(5):e102. doi: 10.2196/jmir.3637 (PMC4468595; doi:10.2196/jmir.3637)

## Slide 1
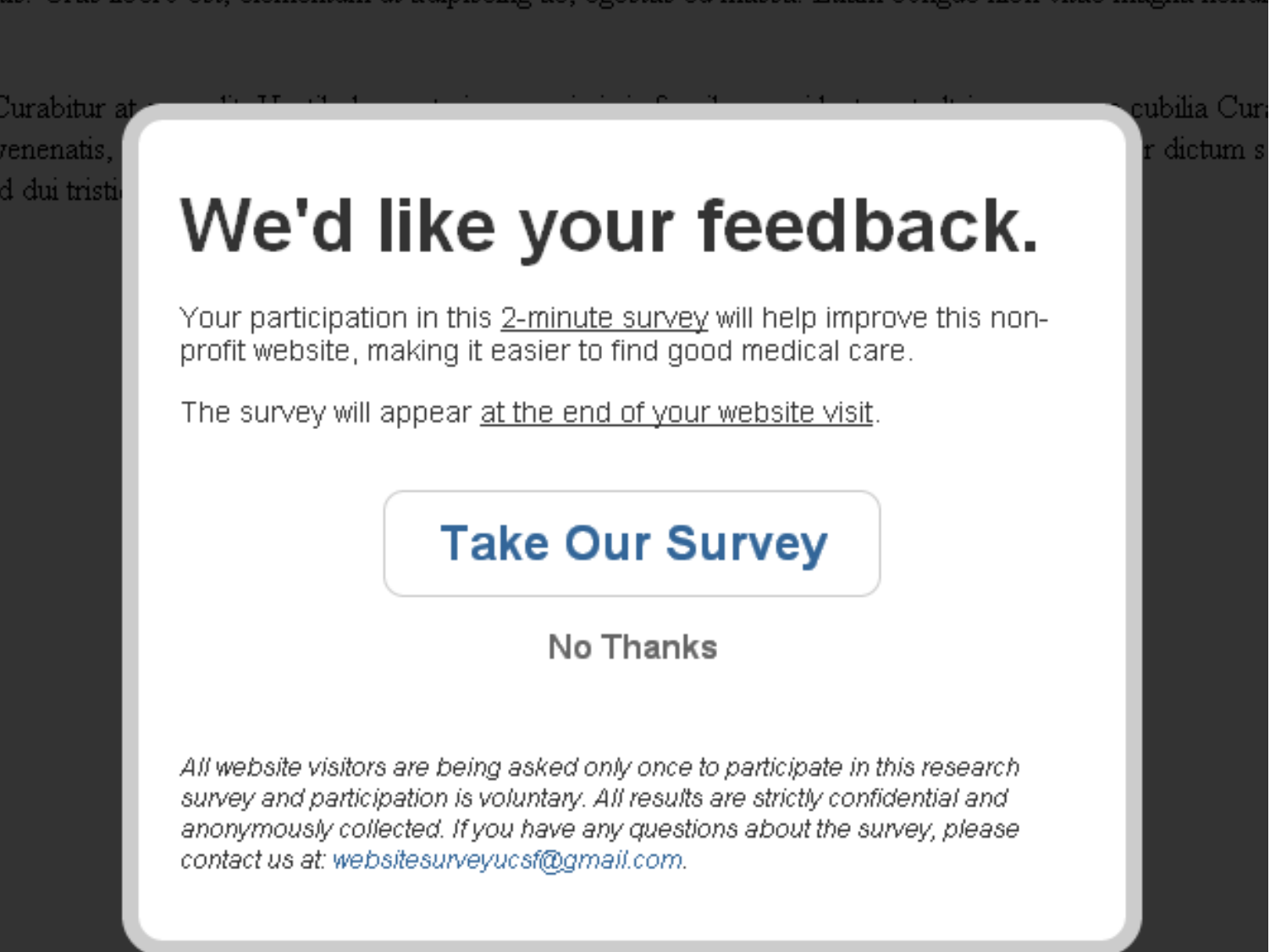

#

## Slide 2
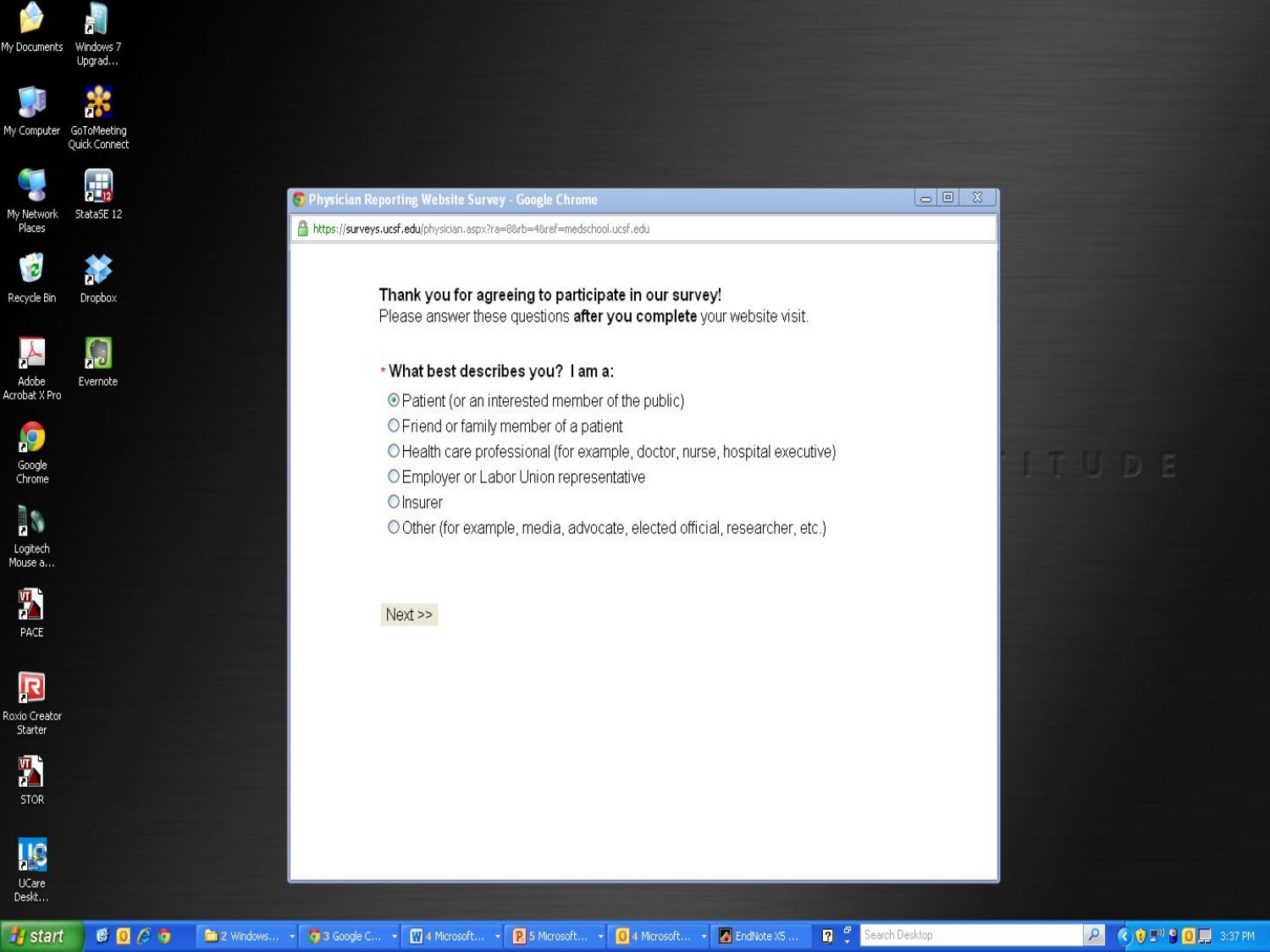

## Slide 3
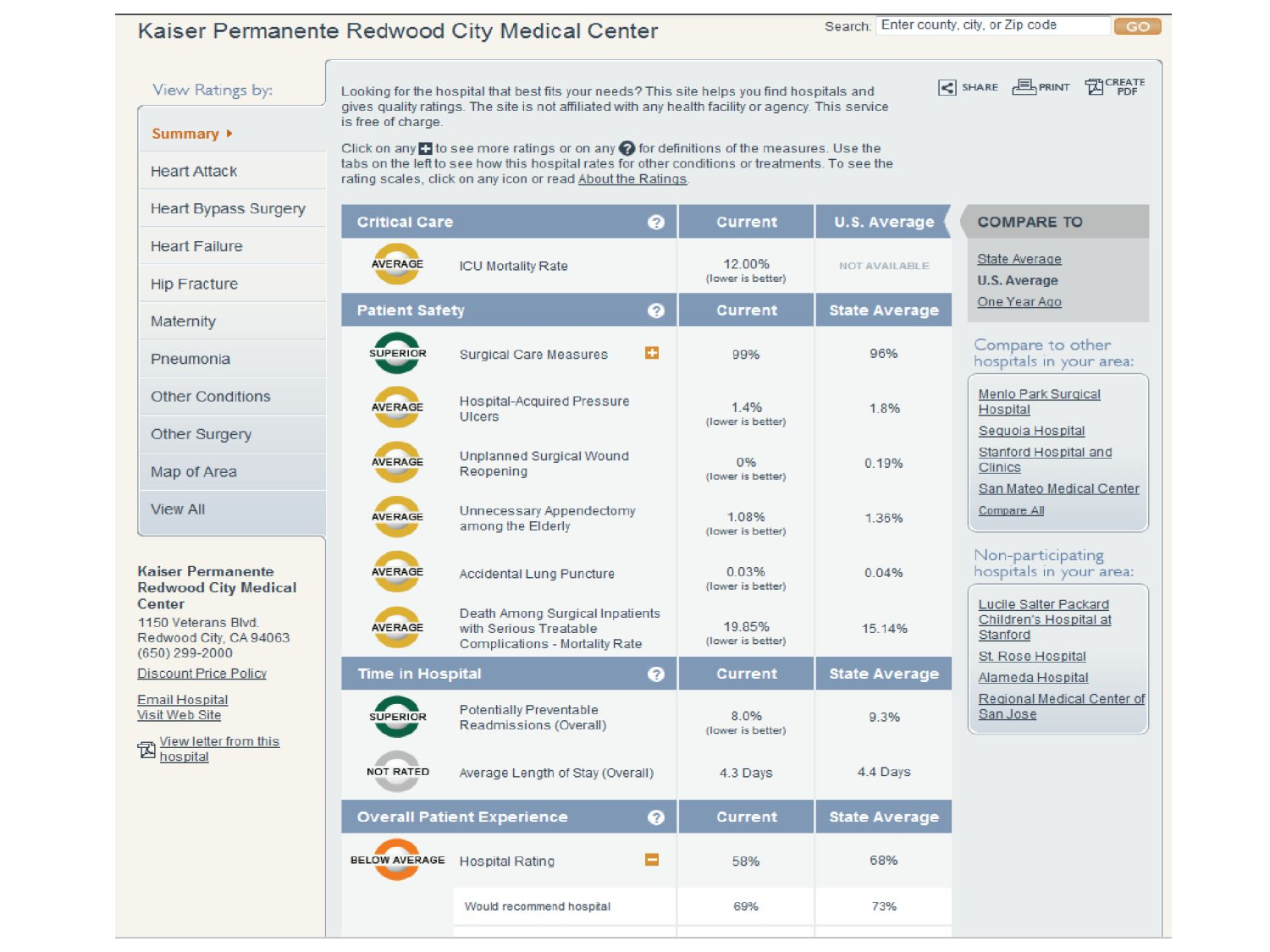

Supplement: Supplementary file 1 [file jmir_v17i5e102_app1.pptx]
