# Supplementary material for: Sources of Traffic and Visitors’ Preferences Regarding Online Public Reports of Quality: Web Analytics and Online Survey Results
Source: J Med Internet Res. 2015 May 1;17(5):e102. doi: 10.2196/jmir.3637 (PMC4468595; doi:10.2196/jmir.3637)
Supplement: Supplementary file 2 [file jmir_v17i5e102_app2.pdf]

eAppendix 1. CHERRIES Checklist for Reporting Results of Internet E-Surveys

eAppendix 2. Description of Survey Development

eAppendix 3. AHRQ Hospital-Public Report Survey

eAppendix 4. AHRQ Physician-Public Report Survey

eAppendix 5. Response Rate Description

eAppendix 1. CHERRIES Checklist for Reporting Results of Internet E-Surveys

**Checklist Item and Explanations**	Relevant Study Information
	**Design**
Describe target population, sample frame. Is the sample a convenience sample? (In "open" surveys this is most likely.)	See Methods section in main text for description of target population. We used an "open survey" design, in which all visitors were offered the opportunity to take the survey if they viewed one of the pages where the survey was programmed to appear. This resulted in a convenience sample of survey respondents.
	**IRB (Institutional Review Board) approval and informed consent process**
Mention whether the study has been approved by an IRB.	This study was approved by the University of California San Francisco Committee on Human Research.
Describe the informed consent process. Where were the participants told the length of time of the survey, which data were stored and where and for how long, who the investigator was, and the purpose of the study?	In the invitation to take the survey (see below in "Contact Mode"), participants were told the length of time and the purpose of the study.<br><br>The purpose of the study was described more specifically at the end of the survey, when the investigators' email was also provided.<br><br>Information was not shared regarding how long the data would be stored nor for how long.
If any personal information was collected or stored, describe what mechanisms were used to protect unauthorized access.	No personal information was collected beyond non-identifying demographics. Survey data were stored on a secure server at UCSF.
	**Development and pre-testing**
Development and testing:	See **eAppendix5** below.

State how the survey was developed, including whether the usability and technical functionality of the electronic questionnaire had been tested before fielding the questionnaire.	
**Recruitment process and description of the sample having access to the questionnaire**	
Open survey versus closed survey? An "open survey" is a survey open for each visitor of a site, while a closed survey is only open to a sample which the investigator knows (password-protected survey).	Open survey
Contact mode: Indicate whether or not the initial contact with the potential participants was made on the Internet. (Investigators may also send out questionnaires by mail and allow for Web-based data entry.)	Only on the Internet. The invitation to take the survey appeared in a pop-up window, and the visitor then was directed to take the survey at the end of the session. The wording of the invitation was:<br><br>"We'd like your feedback.<br><br>Your participation in this 2-minute survey will help improve this nonprofit Web site, making it easier for everyone to find a good hospital or doctor.<br><br>The survey will appear at the end of your Web site visit.<br><br>Take Our Survey No Thanks<br><br>All Web site visitors are being asked only once to participate in this research survey and participation is voluntary. All results are strictly confidential and anonymously collected. If you have any questions about the survey, please contact us at: XXX@xxxx.edu"

How/where was the survey announced or advertised? Some examples are offline media (newspapers), or online (mailing lists – If yes, which ones?) or banner ads (Where were these banner ads posted and what did they look like?). It is important to know the wording of the announcement as it will heavily influence who chooses to participate. Ideally the survey announcement should be published as an appendix.	No advertising was done for the survey.
**Survey administration**	
State the type of e-survey (eg, one posted on a Web site, or one sent out through e-mail).	Web survey
Describe the Web site (for mailing list/newsgroup) in which the survey was posted. What is the Web site about, who is visiting it, what are visitors normally looking for? Discuss to what degree the content of the Web site could pre-select the sample or influence the results. For example, a survey about vaccination on a anti-immunization Web site will have different results from a Web survey conducted on a government Web site	See the Background section of the main text of the paper for a description of the participating Chartered Value Exchange websites. See the Limitations section of the main text for a discussion of potential response bias.
Was it a mandatory survey to be filled in by every visitor who wanted to enter the Web site, or was it a voluntary survey?	Voluntary
Were any incentives offered?	No incentives were offered

In what timeframe were the data collected?	Data were collected February –August 2011
Randomization of items or questionnaires	To decrease cognitive burden, each consumer (patient or friend/family member) was asked only a randomly chosen subset of categories. Questions were always presented in the same order.
Used adaptive questioning (certain items, or only conditionally displayed based on responses to other items) to reduce number and complexity of the questions?	Different items were used for different groups of respondents (patients, friends or family members, healthcare professionals), with respondent type determined in the first page of questions.<br><br>In addition, patients and friends or family members had a larger number of survey items that were relevant to them compared to the other respondent groups. In order to reduce the response burden for them, each patient or firend/family member was shown a random selection of three of five blocks of questions.
What was the number of questionnaire items per page? The number of items is an important factor for the completion rate.	Range 1-9. Most pages had 1-4 items.
Number of screens (pages). Over how many pages was the questionnaire distributed? The number of items is an important factor for the completion rate.	The number of screens ranged from 4-6, depending on the respondent type. At the top of each screen, progress through the survey was reported ("30% complete", next screen "40% complete").
Completeness check. It is technically possible to do consistency or completeness checks before the questionnaire is submitted. Was this done, and if "yes", how (usually JAVAScript)? An alternative is to check for completeness after the questionnaire has been submitted (and highlight mandatory items). If this has been done, it should be reported. All items should	One question was required in order to advance through the rest of the survey (the initial respondent type item). No other questions were mandatory. All non-scaled items had a non-response or "other" option.

provide a non-response option such as “not applicable” or “rather not say”, and selection of one response option should be enforced.	
Review step. State whether respondents were able to review and change their answers (eg, through a Back button or a Review step which displays a summary of the responses and asks the respondents if they are correct).	Respondents were able to change their responses using a “Back” button at the bottom of each screen.
**Response rates**	
Definition of unique site visitor. If you provide view rates or participation rates, you need to define how you determined a unique visitor. There are different techniques available, based on IP addresses or cookies or both.	The denominator for the survey was based on the number of unique visitors who were invited to take the survey. Once the survey invitation had been shown once, it was not shown again on the same computer, using cookies to identify that the survey had been launched.
View rate (Ratio of unique survey visitors/unique site visitors). Requires counting unique visitors to the first page of the survey, divided by the number of unique site visitors (not page views!). It is not unusual to have view rates of less than 0.1 % if the survey is voluntary.	For the numerator of View Rate, we used the number of unique visitors shown the invitation because the first page of survey items was not viewed until a visitor agreed to take the survey. The survey view rate was relatively high, as the survey was designed to survey most website visitors.
Participation rate (Ratio of unique visitors who agreed to participate/unique first survey page visitors). Count the unique number of people who filled in the first survey page (or agreed to participate, for example by checking a checkbox), divided by visitors	As numerator, we counted the number of respondents who completed the first survey question. The denominator was the number of unique visitors shown the invitation (the numerator as described above in View Rate).

who visit the first page of the survey (or the informed consents page, if present). This can also be called “recruitment” rate.	
Completion rate (Ratio of users who finished the survey/users who agreed to participate). The number of people submitting the last questionnaire page, divided by the number of people who agreed to participate (or submitted the first survey page). This is only relevant if there is a separate “informed consent” page or if the survey goes over several pages. This is a measure for attrition. Note that “completion” can involve leaving questionnaire items blank. This is not a measure for how completely questionnaires were filled in. (If you need a measure for this, use the word “completeness rate”.)	We did not calculate the completion rate. Because consumer respondents were randomized to be shown only a specific set of questions, it is difficult to determine whether a blank question is because a consumer was not shown the question or because they did not complete it. Therefore, it is difficult to know where the consumers stopped answering questions and where they stopped being shown questions. The coding of the answers in the survey software did not differentiate between these two types of blanks.
**Preventing multiple entries from the same individual**	
Cookies used? Indicate whether cookies were used to assign a unique user identifier to each client computer. If so, mention the page on which the cookie was set and read, and how long the cookie was valid. Were duplicate entries avoided by preventing users access to the survey twice; or were duplicate database entries having the same user ID eliminated before analysis? In the latter case, which entries were kept for analysis (eg, the	Cookies were used. Once the survey invitation was deployed for an IP address, it did not deploy again. We did not prevent duplicate entries by tracking user access beyond the IP address.

first entry or the most recent)?	
IP check<br>Indicate whether the IP address of the client computer was used to identify potential duplicate entries from the same user. If so, mention the period of time for which no two entries from the same IP address were allowed (eg, 24 hours).	The IP address of the client computer was used to identify potential duplicate entries from the same user. No two entries from the same IP were allowed for the duration of the study period, accomplished by not inviting users with the same IP address to take the survey more than once.
Log file analysis<br><br>Indicate whether other techniques to analyze the log file for identification of multiple entries were used. If so, please describe.	We did not analyze the log file to identify multiple entries. We used the log files to exclude answers that we had tagged as “test” answers using the free text options.
Registration<br><br>In “closed” (non-open) surveys, users need to login first and it is easier to prevent duplicate entries from the same user. Describe how this was done. For example, was the survey never displayed a second time once the user had filled it in, or was the username stored together with the survey results and later eliminated? If the latter, which entries were kept for analysis (eg, the first entry or the most recent)?	Not applicable, since we used an “open” survey design.
**Analysis**	
Handling of incomplete questionnaires<br><br>Were only completed	All surveys were analyzed.

questionnaires analyzed? Were questionnaires which terminated early (where, for example, users did not go through all questionnaire pages) also analyzed?	
Questionnaires submitted with an atypical timestamp<br><br>Some investigators may measure the time people needed to fill in a questionnaire and exclude questionnaires that were submitted too soon.	We did not exclude surveys based on atypical timestamps.
Statistical correction<br><br>Indicate whether any methods such as weighting of items or propensity scores have been used to adjust for the non-representative sample; if so, please describe the methods.	No weighting scheme was used for the analysis of results.

eAppendix 2. Survey Development

We drafted the AHRQ Hospital-Public Report survey (see **eAppendix 3**) based on authors' expert knowledge about online public reports and based on existing online surveys from participating public reporting websites. Participating report sponsors subsequently vetted the survey, giving feedback during a webinar for this purpose, as well as over email. We performed cognitive interviews with 11 potential respondents (including consumers, health care professionals, an employer, and an insurer) in order to improve interpretability of the survey questions and response options. We created the Physician-Public Report survey (see **eAppendix 4**) by making minor modifications to the Hospital survey while maintaining the essential content, and performed cognitive interviews for the new survey with two consumers and two healthcare professionals. The usability and technical functionality of both surveys were tested prior to implementation by research staff and during the cognitive interviews.

eAppendix 3. AHRQ Hospital-Public Report Survey

Survey formatting notes:

- Survey directions are in blue (i.e. anything blue is not visible in survey)
- “Page x” refers to a new survey page
- The survey is programmed to show Page 1 and Page 2 to all respondents. After that, the survey branches to separate question sets for each person-type on Page1. Each Person-type specific question set starts with a new header and new page in the document below.
- For Patients and Friend or Family Members, not all survey pages will be shown. The survey software will show only 3 of the 5 question topics (Purpose of Visit, Topics of Interest, Using the Website, What would you like added or changed, Demographic questions), and is programmed to choose at random which of the three pages to show.

Survey invitation [pops up upon arrival at the website]

We’d like your feedback.

Your participation in this 2-minute survey will help improve this non-profit website, making it easier for everyone to find a good hospital.

The survey will appear at the end of your website visit.

Take Our Survey No Thanks

All website visitors are being asked only once to participate in this research survey and participation is voluntary. All results are strictly confidential and anonymously collected. If you have any questions about the survey, please contact us at: websitesurveyucsf@gmail.com

If a visitor agrees to take the survey by clicking on “Take our survey”, the survey window opens quietly behind the open browser window and the visitor can continue the visit without further interruption.

The survey window remains open to be completed later.

Page 1

Thank you for agreeing to participate in our survey!

Please answer these questions **after you complete** your website visit.

What best describes you? I am a:

Patient (or an interested member of the public) [branch to Patient questions]

Friend or family member of a patient [branch to Friend or Family questions]

Health care professional (for example, doctor, nurse, hospital executive) [branch to Health Care professional questions]

Employer or Labor Union representative [branch to Employer or Labor Union questions]

Insurer [branch to Patient questions]

Other (for example, media, advocate, elected official, researcher, etc.) [branch to Other questions]

Page 2

20% complete

Item 1

How would you rate your experience using the website?

Poor Excellent

0 1 2 3 4 5 6 7 8 9 10

Item 2

How easy was it to use the website?

(for example, not too many clicks, easy to search the website)

Very Difficult Very Easy

0 1 2 3 4 5 6 7 8 9 10

Patient (or interested member of the public)

Page 3

30% complete

Title: **Purpose of your visit**

Item 1

What was the primary purpose of your visit to the website today? To:

(choose only one)

Choose the best hospital for myself or compare hospitals

See how good my hospital is

Prepare for a talk with my doctor

Get information for my family or friends [redirect to Family and Friends survey items]

Learn about a particular disease

Get practical information about a hospital (phone number, location)

Find out how much I would have to pay at a hospital

Other:

Item 2

Were you able to accomplish the primary purpose of your visit?

Definitely

Mostly

Only partially

Not at all

Item 3

Did you have other reasons (in addition to the primary reason above) for your visit today? To:

(choose all that apply)

Choose the best hospital for myself or compare hospitals

See how good my hospital is

Prepare for a talk with my doctor

Get information for my family and friends

Learn about a particular disease

Get practical information about a hospital (phone number, location)

Find out how much I would have to pay at a hospital

No, I had no other reason

Other:

Page 4

40% complete

Title: **Topics of interest to you**

Item 1

What medical conditions are you looking for information about today?
(choose all that apply)
Heart disease (for example, heart attacks or heart failure)
Surgery (for example, hip or knee replacement, or gallbladder surgery)
Obstetrics and gynecology (for example, having a baby)
Pediatric conditions
Cancer
Asthma
Pneumonia
None of the above
Other:

Item 2

What types of information about hospital care are you looking for today?
(choose all that apply)
How often complications or errors occur
How often infections occur
How patients rate their care (for example, how many patients would recommend the hospital)
How often patients survive
How often patients get the correct care (for example, how many stroke patients get the right medicine)
None of the above
Other:

Page 5

50% complete

Title: **Using information from the website**

Item 1

How likely are you to use the website information now to choose a hospital or change hospitals?

Very likely
Likely
Unlikely [branch to page 6]
Very unlikely [branch to page 6]

Item 2
How likely are you to use the website information to have a conversation with your doctor or other health care provider?
Very likely
Likely
Unlikely
Very unlikely

If you searched using Google (or Yahoo, Bing, or other search engine) to get to the website, did you search for a:
Specific hospital (for example, "St. Francis Medical Center")
Website comparing hospitals
Medical problem or treatment (for example, "heart failure" or "high blood sugar")
I did not use Google or another search engine
I do not know
Other:

Page 6
55% complete

Item 1
Why are you unlikely to use the information to choose a hospital?
(choose all that apply)
I do not have a choice of a hospital
I do not need to choose a hospital or change hospitals at this time
The information provided is not specific to my personal health condition
Other factors are more important in my decision-making
The information provided does not cover the specific hospital I want to know about
The information provided did not seem trustworthy
The information is confusing or difficult to understand
Other:

Page 7
75% complete
Title: **What would you like added or changed?**

Item 1

What additional information would make the website more useful? More information about: (choose all that apply)

How well the hospital treats my specific medical condition
How well the hospital does my specific surgery or procedure
How much I would pay
Practical aspects of the hospital (for example, phone number, location)
Comments from people who have been patients at the hospital
Hospitals that are not currently on the website
Individual doctors within a hospital
Other:

Item 2

What would make the website easier to use for you?
(choose all that apply)

Provide a different way of searching the website
Provide a different way of sorting the results on the website
Make the information easier to understand (for example, less technical terms)
Make it easier to find the best hospital
Require fewer clicks to get information that I want
Other:

Item 3

Please use this box to add details about your choices in the prior two questions or comments about how to make the website more useful to you. [Free text box]

Item 4

How likely are you to visit the website again?

Very likely
Likely
Unlikely
Very unlikely

Page 8

85% complete

Title: A few last questions about you

Item 1

What is your age?
<18 years old
18 to 24
25 to 34
35 to 44
45 to 54
55 to 64
65 to 74
75 or older

Item 2

Are you male or female?
Male
Female

Item 3

Are you of Hispanic or Latino descent?
Yes, Hispanic or Latino
No, not Hispanic or Latino

Item 4

What is your race?
White
Black or African-American
Asian
Native Hawaiian or other Pacific Islander
American Indian or Alaska Native
Other

Item 5

What is the highest grade or level of school that you have completed?
8th grade or less
Some high school, but did not graduate
High school graduate or GED
Some college or 2-year degree
4-year college graduate
More than 4 year-college degree

Item 6

What type of health care insurance do you have?

(choose only one)
Private insurance (i.e. from your employer or purchased by you)
Medicare
Medicaid
No insurance
I don’t know
Other:

Health Care Professional

Page 9

40% complete

Title: **Purpose of your visit**

Item 1

What was the primary purpose of your visit to the website today? To:

(choose only one)

See how my hospital is performing

Compare my hospital's performance to other hospitals' performance

Choose a hospital to make a patient referral

Choose a hospital for myself [bounce to "HCP Patient or might be patient" items at end of survey]

Get practical information about a hospital (phone number, location)

Other:

Item 2

Were you able to accomplish the primary purpose of your visit?

Definitely

Mostly

Only partially

Not at all

Item 3

Did you have other reasons (in addition to the primary reason above) for your visit today? To:

(choose all that apply)

See how my hospital is performing

Compare my hospital's performance to other hospitals' performance

Choose a hospital for the purposes of making a patient referral

Choose a hospital for myself

Get practical information about a hospital (phone number, location)

No, I had no other reason

Other:

Page 10

60% complete

Title: **What would you like added or changed?**

Item 1

What additional information would make the website more useful? More information about: (choose all that apply)

Quality measures that are not currently included

Quality by inpatient service (for example, intensive care unit, pediatric unit)

Costs for patients (for example, cost of a procedure, cost of a hospital stay)

Methodology used to calculate performance measures (for example, risk adjustment methods)

Detailed results for each hospital (for example, sample size or 95% confidence intervals around their performance)

Hospitals that are not currently on the website

Individual doctors within a hospital

Other:

Item 2

What would make the website easier to use for you?

(choose all that apply)

Provide a different way of searching the website

Provide a different way of sorting the results on the website

Make the information easier to understand (for example, less technical terms)

Make it easier to find the best hospital

Require fewer clicks to get information that I want

Other:

Item 3

Please use this box to add details about your choices in the prior two questions or comments about how to make the website more useful to you. [Free text box]

Item 4

How likely are you to visit the website again?

Very likely

Likely

Unlikely

Very unlikely

80% complete

Title: **A few last questions about you**

Item 1
Are you a:
(choose all that apply)
Physician [branch to page 35, for specialty question]
Nurse practitioner [branch to page 35, for specialty question]
Pharmacist
Social worker
Executive
Quality manager
Other:

Item 2
What is your age?
18 to 24
25 to 34
35 to 44
45 to 54
55 to 64
65 to 74
75 or older

Item 3
What is the zip code at your primary workplace?

If you searched using Google (or Yahoo, Bing, or other search engine) to get to the website, did you search for a:
Specific hospital (for example, “St. Francis Medical Center”)
Website comparing hospitals
Medical problem or treatment (for example, “heart failure” or “high blood sugar”)
I did not use Google or another search engine
I do not know
Other:

Friend or Family Member

Page 12

30% complete

Item 1

I am looking for information for a:

(choose all that apply)

Family member

Non-family member

Child (under 18 years)

Non-senior adult (18-65 years)

Senior (65+)

Non-English speaker. Please specify language spoken:

Page 13

40% complete

Title: **Purpose of your visit**

Item 1

What was the primary purpose of your visit to the website today? To:

(choose only one)

Choose the best hospital or compare hospitals

See how good a specific hospital is

Prepare for a talk with a doctor

Learn about a particular disease

Get practical information about a hospital (phone number, location)

Find out how much one would have to pay at a hospital

Other:

Item 2

Were you able to accomplish the primary purpose of your visit?

Definitely

Mostly

Only partially

Not at all

Item 3

Did you have other reasons (in addition to the primary reason above) for your visit today?
To:
(choose all that apply)
Choose the best hospital or compare hospitals
See how good a specific hospital is
Prepare for a talk with a doctor
Learn about a particular disease
Get practical information about a hospital (phone number, location)
Find out how much one would have to pay at a hospital
No, I had no other reason
Other:

Page 14

50% complete

Title: **Topics of interest to you**

Item 1
What medical conditions are you looking for information about today?
(choose all that apply)
Heart disease (for example, heart attacks or heart failure)
Surgery (for example, hip or knee replacement, or gallbladder surgery)
Obstetrics and gynecology (for example, having a baby)
Pediatric conditions
Cancer
Asthma
Pneumonia
None of the above
Other:

Item 2
What types of information about hospital care are you looking for today?
(choose all that apply)
How often complications or errors occur
How often infections occur
How patients rate their care (for example, how many patients would recommend the hospital)
How often patients survive

How often patients get the correct care (for example, how many stroke patients get the right medicine)
None of the above
Other:

Item 3
What type of health care insurance does your friend or family member have?
(choose one)
Private insurance (i.e. from their employer or purchased by them)
Medicare
Medicaid
No insurance
I don't know
Other:

Page 15
60% complete

Title: **Using information from the website**

Item 1
How likely are you to use the information from the website now to help your friend or family member to choose a hospital or change hospitals?
Very likely
Likely
Unlikely [branch to page 16]
Very unlikely [branch to page 16]

Item 2
How likely are you or your friend or family member to use the information from the website to have a conversation with a doctor or other health care provider?
Very likely
Likely
Unlikely
Very unlikely

If you searched using Google (or Yahoo, Bing, or other search engine) to get to the website, did you search for a:
Specific hospital (for example, "St. Francis Medical Center")
Website comparing hospitals

Medical problem or treatment (for example, “heart failure” or “high blood sugar”)
I did not use Google or another search engine
I do not know
Other:

Page 16

65% complete

Item 1

Why are you unlikely to use the information to help your friend or family member choose a hospital?
(choose all that apply)
My friend or family member does not have a choice of a hospital
He or she does not need to choose a hospital or change hospitals at this time
The information provided is not specific to his or her personal health condition
Other factors are more important in the decision-making
The information provided does not cover the specific hospital he or she needs to know about
The information provided did not seem trustworthy
The information is confusing or difficult to understand
Other:

Page 17

80% complete

Title: **What would you like added or changed?**

Item 1

What additional information would make the website more useful? More information about:
(choose all that apply)
How well the hospital treats the specific medical condition my friend or family member has
How well the hospital does the specific surgery or procedure my friend or family member needs
How much my friend or family member would pay (for example, cost of a procedure or cost of a hospital stay)
Practical aspects of the hospital (for example, phone number, location)
Comments from people who have been patients at the hospital
Hospitals that are not currently on the website
Individual doctors within a hospital
Other:

Item 2
What would make the website easier to use for you?
(choose all that apply)
Provide a different way of searching the website
Provide a different way of sorting the results on the website
Make the information easier to understand (for example, less technical terms)
Make it easier to find the best hospital
Require fewer clicks to get information that I want
Other:

Item 3
Please use this box to add details about your choices in the prior two questions or comments about how to make the website more useful to you. [Free text box]

Item 4
How likely are you to visit the website again?
Very likely
Likely
Unlikely
Very unlikely

Page 18
90% complete

Title: **A few last questions about YOU**

Item 1
What is *your* age?
<18 years old
18 to 24
25 to 34
35 to 44
45 to 54
55 to 64
65 to 74
75 or older

Item 2
Are *you* male or female?
Male

Female

Item 3

What is the highest grade or level of school that *you* have completed?

8th grade or less

Some high school, but did not graduate

High school graduate or GED

Some college or 2-year degree

4-year college graduate

More than 4 year-college degree

Page 19

Completion Event

Thank you for taking the survey!

Your participation will help us understand how to make the website better so that people can easily find information about how well hospitals take care of their patients.

The study was conducted by Dr. R. Adams Dudley's research team at the Philip R. Lee Institute for Health Policy Studies, at the University of California San Francisco.

If you have any questions about the survey, please contact us at: websitesurveyucsf@gmail.com

You may now return to http://participatingwebsitehere.org or simply close this window.

eAppendix 4. Physician-Public Report Survey

Survey formatting notes:

- Survey directions are in blue (i.e. anything blue is not visible in survey)
- “Page x” refers to a new survey page
- The survey is programmed to show Page 1 and Page 2 to all respondents. After that, the survey branches to separate question sets for each person-type on Page1. Each person-type specific question set starts with a new header and new page in the document below.
- Randomization to decrease response burden: For Patients (including Health Care Professionals who indicate that they are “Choosing a @@092393 for myself”) and Friend or Family Members, not all survey pages will be shown. The survey software will show only 3 of the 5 question topics (Purpose of Visit, Topics of Interest, using the Website, What would you like added or changed, Demographic questions), and is programmed to choose at random which of the three pages to show.
- Piping in: The answer to Item 1 on Page 2 is used throughout the rest of the survey wherever @@092393 appears.

Survey invitation [pops up upon arrival at the website]

We’d like your feedback.
Your participation in this 2-minute survey will help improve this non-profit website, making it easier to find good medical care.

The survey will appear at the end of your website visit.

Take Our Survey No Thanks

All website visitors are being asked only once to participate in this research survey and participation is voluntary. All results are strictly confidential and anonymously collected. If you have any questions about the survey, please contact us at: websitesurveyucsf@gmail.com.

If a visitor agrees to take the survey by clicking on “Take our survey”, the survey window opens quietly behind the open browser window and the visitor can continue the visit without further interruption.
The survey window remains open to be completed later.

Page 1

Thank you for agreeing to participate in our survey!
Please answer these questions **after you complete** your website visit.

* What best describes you? I am a:
Patient (or interested member of the public)
Friend or family member of a patient
Health care professional (for example, doctor, nurse, hospital executive)
Employer or Labor Union representative
Insurer
Other (for example, media, advocate, elected official, researcher, etc.)

Page 2

20% complete

Item 1
* Are you looking for information about a doctor, a clinic, or a medical group?
A “clinic” is an office with multiple doctors all in the same location.
A “medical group” is a large network of doctors who do not necessarily work in the same location.
doctor
clinic
medical group
other:

Item 2
How would you rate your experience with using the website?

Poor										Excellent
0	1	2	3	4	5	6	7	8	9	10

Item 3
How easy was it to use the website?
(for example, not too many clicks, easy to search the website)

Very Difficult Very Easy

0	1	2	3	4	5	6	7	8	9	10

HEALTH CARE PROFESSIONAL

Page 3

40% complete

Purpose of your visit

Item 1
What was the primary purpose of your visit to the website today? To:
(choose only one)
See my or my group's performance
See the performance of another physician or other group
Compare my or my group's performance to others' performance
Compare my or my group’s charges to others’ charges
Choose a @@092563 to make a patient referral
Choose a @@092563 for myself [branch to HEALTH CARE PROFESSIONAL CHOOSE A xxx FOR MYSELF]
Get practical information about a @@092563 (phone number, location)
Other:

Item 4
Were you able to accomplish the primary purpose of your visit?
Definitely
Mostly
Only partially
Not at all

Item 5
Did you have other reasons (in addition to the primary reason above) for your visit today?
To:
(choose all that apply)
See my or my group's performance
See the performance of another physician or other group
Compare my or my group’s performance to others’ performance
Choose a @@092563 to make a patient referral
Choose a @@092563 for myself
Get practical information about a @@092563 (phone number, location)
No, I had no other reason
Other:

60% complete

What would you like added or changed?

Item 1

What additional information would make the website more useful? Information about:
(choose all that apply)

Quality measures that are not on the website

A @@092563 that is not on the website

Costs for patients (for example, medication costs or costs of a referral)

Methodology used to calculate performance measures (for example, risk adjustment methods)

Detailed results for each @@092563 (for example, sample size or 95% confidence intervals around their performance)

Individual doctor performance (for example, proportion of the doctor's diabetes patients who got the right test)

No other information

Other:

Item 2

What would make the website easier for you to use?
(choose all that apply)

Provide a different way of searching the website

Provide a different way of sorting the results on the website

Make the information easier to understand (for example, fewer technical terms)

Make it easier to compare @@092393s on one page (for example, show them side by side with only the information needed)

Make it easier to find a @@092393 by location

Make it easier to identify a good @@092393. How?

Item 3

Please list any quality measures or @@092393(s) that you would like to see on the website.

[free text box]

Item 4

Please write any additional comments about how the website could be more useful or easier to use.

[free text box]

Item 5
How likely are you to visit the website again?
Very likely
Likely
Unlikely
Very unlikely

Page 5

80% complete

A few last questions about you

Item 1
Are you a:
(choose all that apply)
Physician [branch to Specialty Question]
Nurse practitioner [branch to Specialty Question]
Pharmacist
Social worker
Executive
Quality manager
Other:

Item 2
In what setting do you primarily work?
Solo or small group practice (less than 10 providers)
Larger group practice (10+ providers)
Hospital
Other:

Item 3
What is your age?

18 to 24
25 to 34
35 to 44
45 to 54
55 to 64
65 to 74
75 or older

Item 4
If you got to the website by using Google (or Yahoo, Bing, or other search engine), did you search for a:
Specific @@092563 (for example, Dr. Smith or Dr. Smith’s clinic)
Type of @@092563 (for example, pediatrician or pediatric clinics)
Website comparing @@092563s
Medical problem or treatment (for example, "high blood sugar" or "cancer surgery")
I did not use Google or another search engine
I do not know
Other:

Item 5
What is the zip code at your primary workplace?

PATIENT (or interested member of the public)

Page 6

30% complete

Purpose of your visit

Item 1

What was the primary purpose of your visit to the website today? To:

(choose only one)

Choose the best @@092563 for myself or compare @@092563s

See how good my @@092563 is

Prepare for a talk with my doctor

Get information for my family or friends

Learn about a particular disease or treatment option

Get practical information about a @@092563 (phone number, location)

Find out how much I would have to pay for care from a specific @@092563

Other:

Item 2

Were you able to accomplish the primary purpose of your visit?

Definitely

Mostly

Only partially

Not at all

Item 3

Did you have other reasons (in addition to the primary reason above) for your visit today? To:

(choose all that apply)

Choose the best @@092563 for myself or compare @@092563s

See how good my @@092563 is

Prepare for a talk with my doctor

Get information for my family or friends

Learn about a particular disease or treatment option

Get practical information about a @@092563 (phone number, location)

Find out how much I would have to pay for care from a specific @@092563

No, I had no other reason

Other:

Page 7

40% complete

Topics of interest to you

Item 1
When you are choosing a @@092563, are there any particular conditions you are concerned about?
(choose all that apply)
Heart disease (for example, high blood pressure or heart failure)
Obstetrics and gynecology (for example, pregnancy)
Cancer
Pediatrics
Asthma
Pneumonia
Diabetes
Other:

Item 2
What specific types of information are you looking for today?
(choose all that apply)
How patients rate their care (for example, how many patients would recommend the @@092563)
How often patients get the correct care (for example, how many people having a heart attack get the right medicine)
How often mistakes occur (for example, medication prescribing mistakes)
Other:

Item 3
What types of doctors are you interested in?
(choose all that apply)
Doctors providing routine pregnancy care
Doctors providing other routine care (for example, preventive or primary care)
Surgeons
Doctors other than surgeons providing specialty care (for example, cancer doctors or heart doctors)

Page 8

50% complete

Using information from the website

Item 1
How likely are you to use the website information now to choose a @@092563 or change @@092563s?
Very likely
Likely
Unlikely [branch to page 33]
Very unlikely [branch to page 33]

Item 2
How likely are you to use the website information to have a conversation with your doctor or other health care provider?
Very likely
Likely
Unlikely
Very unlikely

Item 3
What do you like about the website?
(choose all that apply)
The number of @@092563s included
The range of conditions and treatments included
Information provided about quality of care (for example, whether the right treatments were given)
Information about how to talk to your doctor
Information about how to choose a doctor
Nothing (I did not like anything about the website)
Other:

Item 4
If you got to the website by using Google (or Yahoo, Bing, or other search engine), did you search for a:
Specific @@092563 (for example, Dr. Smith or Dr. Smith’s clinic)
Type of @@092563 (for example, pediatrician or pediatric clinics)
Website comparing @@092563s
Medical problem or treatment (for example, "high blood sugar" or "cancer surgery")
I did not use Google or another search engine

I do not know
Other:

Page 9

55% complete

Item 1
Why are you unlikely to use the information to choose a @@092563?
(choose all that apply)
I do not have a choice of a @@092563
I do not need to choose a @@092563 at this time
The website does not provide information specific to my personal health condition
The website does not provide information specific to the @@092563 that I want to know about
The information provided does not seem trustworthy
The information is confusing or difficult to understand
Other:

Page 10

75% complete

What would you like added or changed?

Item 1
What additional information would make the website more useful? Information about:
(choose all that apply)
How well the @@092563 treats patients with my specific condition
Comments from people who have been the @@092563's patient
A @@092563 that is not on the website
How much I would pay for care from a specific @@092563
Practical information about the @@092563 (for examples, phone number, location)
No other information
Other:

Item 2
What would make the website easier for you to use?
(choose all that apply)
Provide a different way of searching the website

Provide a different way of sorting the results on the website
Make the information easier to understand (for example, fewer technical terms)
Make it easier to compare @@092393s on one page (for example, show them side by side with only the information needed)
Make it easier to find any @@092393 in my neighborhood
Make it easier to identify a good @@092393. How?

Item 3
Please list any additional @@092563(s) you would like to see on the website.

Item 4
Please write any additional comments about how to make the website more useful or easier to use.

Item 5
How likely are you to visit the website again?
Very likely
Likely
Unlikely
Very unlikely

Page 11

85% complete

A few last questions about you

Item 1
What is your age?
<18 years old
18 to 24
25 to 34
35 to 44
45 to 54
55 to 64
65 to 74
75 or older

Item 2
Are you male or female?

Male
Female

Item 3
Are you of Hispanic or Latino descent?
Yes, Hispanic or Latino
No, not Hispanic or Latino

Item 4
What is your race?
White
Black or African-American
Asian
Native Hawaiian or other Pacific Islander
American Indian or Alaska Native
Other

Item 5
What is the highest grade or level of school that you have completed?
8th grade or less
Some high school, but did not graduate
High school graduate or GED
Some college or 2-year degree
4-year college graduate
More than 4 year-college degree

Item 6
What is your primary health insurance plan?
(choose only one)
Private insurance (i.e. from your employer or purchased by you)
Medicare
Medicaid
No insurance
I don't know
Other:

Item 7
What is the zipcode of your primary residence?

Item 8

If you got to the website by using Google (or Yahoo, Bing, or other search engine), did you search for a:

Specific @@092563 (for example, Dr. Smith or Dr. Smith’s clinic)

Type of @@092563 (for example, pediatrician or pediatric clinics)

Website comparing @@092563s

Medical problem or treatment (for example, "high blood sugar" or "cancer surgery")

I did not use Google or another search engine

I do not know

Other:

FRIEND OR FAMILY MEMBER

Page 12

30% complete

Item 1

I am looking for information for a:

(choose all that apply)

Family member

Non-family member

Child (under 18 years)

Non-senior adult (18-65 years)

Senior (65+)

Non-English speaker. Please specify language spoken:

Page 13

Purpose of your visit

Item 1

What was the primary purpose of your visit to the website today? To:

(choose only one)

Choose the best @@092563 or compare @@092563s

See how good a specific @@092563 is

Prepare for a talk with a doctor

Choose a @@092563 for myself

Learn about a particular disease or treatment option

Get practical information about a @@092563 (phone number, location)

Find out how much one would have to pay for care from a specific @@092563

Other:

Item 2

Were you able to accomplish the primary purpose of your visit?

Definitely

Mostly

Only partially

Not at all

Item 3

Did you have other reasons (in addition to the primary reason above) for your visit today? To:

(choose all that apply)

Choose the best @@092563 or compare @@092563s
See how good a specific @@092563 is
Prepare for a talk with a doctor
Learn about a particular disease or treatment option
Get practical information about a @@092563 (phone number, location)
Find out how much one would have to pay for care from a specfic @@092563
No, I had no other reason
Other:

Page 14
50% complete

Topics of interest to you

Item 1
When you are choosing a @@092563 for your friend or family member, are there any particular conditions you are concerned about?
(choose all that apply)
Heart disease (for example, heart attacks or heart failure)
Obstetrics and gynecology (for example, pregnancy)
Cancer
Pediatrics
Asthma
Pneumonia
Diabetes
Other:

Item 2
What specific types of information are you looking for today?
(choose all that apply)
How patients rate their care (for example, how many patients would recommend the @@092563)
How often patients get the correct care (for example, how many people having a heart attack get the right medicine)
How often mistakes occur (for example, medication prescribing mistakes)
Other:

Item 3
What types of doctors are you interested in?
(choose all that apply)

Doctors providing routine pregnancy care
Doctors providing other routine care (for example, preventive or primary care)
Surgeons
Doctors other than surgeons providing specialty care (for example, cancer doctors or heart doctors)

Item 4
What is your friend or family member's primary health insurance plan?
(choose one)
Private insurance (i.e. from their employer or purchased by them)
Medicare
Medicaid
No insurance
I don’t know
Other:

Page 15

60% complete

Using information from the website

Item 1
How likely are you to use the website information now to help your friend or family member choose a @@092563 or change @@092563s?
Very likely
Likely
Unlikely [branch to Page 27]
Very unlikely [branch to Page 27]

Item 2
How likely are you or your friend or family member to use the information from the website to have a conversation with a doctor or other health care provider?
Very likely
Likely
Unlikely
Very unlikely

Item 3
What do you like about the website?

(choose all that apply)
The number of @@092563s included
The type of @@092563s included
The range of conditions and treatments included
Information provided about quality of care (for example, whether the right treatments were given)
Information about how to talk to your doctor
Information about how to choose a doctor
Nothing (I did not like anything about the website)
Other:

Item 4
If you got to the website by using Google (or Yahoo, Bing, or other search engine), did you search for a:
Specific @@092563 (for example, Dr. Smith or Dr. Smith's clinic)
Website comparing @@092563s
Medical problem or treatment (for example, "high blood sugar" or "cancer surgery")
Type of @@092563 (for example, pediatrician or pediatric clinics)
I did not use Google or another search engine
I do not know
Other:

Page 16

65% complete

Item 1
Why are you unlikely to use the information to help your friend or family member choose a @@092563?
(choose all that apply)
My friend or family member does not have a choice of a @@092563
He or she does not need to choose a @@092563 or change @@092563s at this time
The website does not provide information specific to his or her personal health condition
The website does not provide information specific to the @@092563 he or she needs to know about
The information provided does not seem trustworthy
The information is confusing or difficult to understand
Other:

Page 17

80% complete

What would you like added or changed?

Item 1
What additional information would make the website more useful? Information about:
(choose all that apply)
How well the @@092563 treats the specific condition my friend or family member has
Comments from people who have been the @@092563's patient
A @@092563 that is not on the website. Which one?
How much my friend or family member would pay for care from a specific @@092563
Practical aspects of the @@092563 (for example, phone number, location)
Other:

Item 2
What would make the website easier for you to use?
(choose all that apply)
Provide a different way of searching the website
Provide a different way of sorting the results on the website
Make the information easier to understand (for example, fewer technical terms)
Make it easier to compare @@092393s on one page (for example, show them side by side with only the information needed)
Make it easier to find any @@092393 in my neighborhood
Make it easier to identify a good @@092393. How?

Item 3
Please list any additional @@092563(s) you would like to see on the website.

Item 4
Please write any additional comments about how to make the website more useful or easier to use.

Item 5
How likely are you to visit the website again?
Very likely
Likely
Unlikely
Very unlikely

Page 18

90% complete

A few last questions about YOU

Item 1
What is your age?
<18 years old
18 to 24
25 to 34
35 to 44
45 to 54
55 to 64
65 to 74
75 or older

Item 2
Are you male or female?
Male
Female

Item 3
What is the highest grade or level of school that you have completed?
8th grade or less
Some high school, but did not graduate
High school graduate or GED
Some college or 2-year degree
4-year college graduate
More than 4 year-college degree

Item 4
What is the zipcode of your primary residence?

Completion Event

Thank you for taking the survey!

Your participation will help us understand how to make the website better so that people can easily find information about how well doctors and medical groups take care of their patients.

The study was conducted by Dr. R. Adams Dudley's research team at the Philip R. Lee Institute for Health Policy Studies, at the University of California San Francisco.

If you have any questions about the survey, please contact us at:
websitesurveyucsf@gmail.com
You may now return to [referring website] or simply close this window.

eAppendix 5. Response Rate Description

We surveyed participating website visitors from February-August 2011. We used an “open survey” design, in which all visitors were offered the opportunity to take the survey if they viewed pages the report sponsors designated as having relevant quality content. The invitation to take the survey appeared in a pop-up window, and the visitor was directed to take the survey at the end of the session.

Response rate for the survey was based on the number of unique visitors who were invited to take the survey. Once the survey invitation had been shown once, it was not shown again on the same computer, using cookies to identify that the survey had been launched. Because web analytics do not differentiate between whether a person has been to another page on the website, there was a potentially for over-counting of unique visitors if unique visitors to all pages where the survey was launched are counted separately.

Report sponsors varied in which pages (defined as separate URLs) to launch the survey, with some choosing to launch from all pages of the website, some only launching from the home page, and some launching from several different pages. We used the following algorithm to calculate the denominator for each website, based on website pages the survey was launched from: if all pages, we used the total number of unique visitors; if only one page, we used the count of unique visitors only to that page; if multiple URLs, including the home page, we used the count of unique visitors to the home page; if multiple pages, without the home page, we used the count of unique visitors from the multiple URLs.
