# Supplementary material for: Sources of Traffic and Visitors’ Preferences Regarding Online Public Reports of Quality: Web Analytics and Online Survey Results
Source: J Med Internet Res. 2015 May 1;17(5):e102. doi: 10.2196/jmir.3637 (PMC4468595; doi:10.2196/jmir.3637)
Supplement: Supplementary file 3 [file jmir_v17i5e102_app3.pdf]

eTable 1. Description of Website Survey Respondents

	**Respondents (N=1755)[a]**	**Persons in catchment areas reporting using internet to research plans or practitioners, percent [b]**
**Consumers, n (% all respondents)**	850 (48.4)	--
Type of Consumer, n (% all respondents)		
Patient	709 (40.4)	--
Friend or Family Member	141 (8.0)	--
Age groups (n=442 shown question) n (% non-missing)		
>18 to 34	27 (8.1)	25.2
35 to 44	38 (11.4)	22.3
45 to 64	194 (58.1)	39.4
65 and older	75 (22.5)	12.6
Female (n= 442 shown question) n (% non-missing)	209 (63.3)	57.6
Education (n= 442 shown question) n (% non-missing)		
High School or GED	24 (7.3)	15.1
Some college	88 (26.7)	30.6
4-year college degree or higher	218 (66.1)	50.9
Race (n= 442 shown question) n (% non-missing)		
White	217 (84.4)	83.1
Black	4 (1.6)	7.2
Asian	12 (4.7)	8.6
Other	36 (14.0)	1.1
Hispanic (n= 442 shown question) (n, % non-missing)	9 (3.4)	9.3
Patient Insurance (n, %)[b] (n= 441 non-missing)		
Private	238 (74.1)	--
Medicare	61 (19.0)	--
Medicaid	5 (1.6)	--
None	10 (3.1)	--
Other or Unknown	7 (2.2)	--
**Healthcare Professionals** n (% all respondents)	551 (31.4)	--
Type of Healthcare Professional (n= 433 shown question) n (% non-missing)		--
Doctors	45 (18.5)	--

Nurse Practitioners or Nurses	65 (26.8)	--
Quality Managers or Other Healthcare Professional	62 (25.5)	--
Executives	71 (29.2)	--
Age groups (n= 433 shown question) n (% non-missing)		
25 to 34	29 (8.7)	--
35 to 44	33 (9.9)	--
45 to 54	120 (35.9)	--
55 to 64	134 (40.1)	--
65 and older	18 (5.4)	--
**Other[c] n (% all respondents)**	354 (19.8)	--

[a] Respondents were required to answer the person-type question ("Who are you? Patient, Friend or family member, Healthcare Professional," etc.), so there are no missings for the bolded person-type categories.
[b] Data from US Census Bureau using the 2011 Internet Supplement,[16] from the catchment areas of the websites.
[c] Patient insurance type is according to the patient response, or according to responses from the friend or family member using the website on behalf of a patient.
[d] Other includes "Employer or Labor Union," "Insurer," "Researcher," "Media," "Lawyer," "Legislator" and a free-text option.
